# Anticoagulant versus Antiplatelet Therapy After Acute Coronary Syndromes in Patients with Coronary Artery Ectasia: A Retrospective Cohort Study

**DOI:** 10.1007/s10557-025-07784-0

**Published:** 2025-09-24

**Authors:** Fabio Solis-Jiménez, Ximena Latapi-Ruiz Esparza, Hannah Priscila Guzman-Solorzano, Monserrat Villalobos-Pedroza, Luis Angel Morales-Villamil, Braiana Angeles Diaz-Herrera, Sarai Hernandez-Pastrana, Rodrigo Gopar-Nieto, Eduardo A. Arias-Sanchez, Luis Alfonso Marroquín-Donday, Gian Manuel Jiménez-Rodríguez, Daniel Sierra-Lara, Diego Araiza-Garaygordobil, Alexandra Arias-Mendoza

**Affiliations:** 1 Universidad Nacional Autónoma de México, Mexico City, Mexico; 2 Facultad de Medicina de la Universidad de Colima, Colima, Mexico; 3 Benemerita Universidad Autonoma de Puebla, Puebla, Mexico; 4 Interventional Cardiology Department, Instituto Nacional de Cardiologia Ignacio Chávez, Mexico City, Mexico; 5 Coronary Care Unit Department, Instituto Nacional de Cardiología Ignacio Chávez, Mexico City, Mexico

**Keywords:** Coronary artery ectasia, Acute coronary syndromes, Myocardial infarction, Anticoagulation, DAPT, Antithrombotic therapy

## Abstract

**Purpose:**

Patients with coronary ectasia (CAE) have an increased risk of major cardiovascular events (MACE). Current preventive treatments are uncertain, with oral anticoagulants often prescribed based on limited retrospective studies. Our aim is to help address the question: what is the most appropriate treatment?

**Methods:**

Using a retrospective cohort of patients with an ACS and CAE in a single center in Mexico City, two groups were observed based on the treatment at discharge: dual antiplatelet therapy (group 1) and anticoagulation with either a VKA or a DOAC, regardless of antiplatelet therapy (group 2). The main outcome was MACE, which was a composite of all-cause mortality, reinfarction, and ischemic stroke at 4.5 years follow-up.

**Results:**

A total of 354 patients admitted for ACS and CAE were included. 228 (64.4%) patients were classified in the DAPT group and 126 (35.5%) in the anticoagulants group. The DAPT group had higher type 2 diabetes rates, NSTEMI presentation, and lower-grade ectasia. The anticoagulation group had higher STEMI presentation and higher-grade ectasia. The DAPT group had 33 (14.5%) events of MACE, whereas the anticoagulation group had 16 (13.1%) events. Anticoagulants were not associated with a risk reduction of the primary endpoint (HR 0.95; 95% CI, 0.47–1.54; *p = *0.59), nor any of the individual components.

**Conclusion:**

This retrospective cohort study showed similar effectiveness between DAPT and anticoagulation in patients with ACS and CAE for preventing MACE, and lower bleeding risk. Further research is needed to identify optimal candidates for each antithrombotic regime.

**Graphical Abstract:**

DAPT: double antiplatelet therapy, HR: hazard ratio

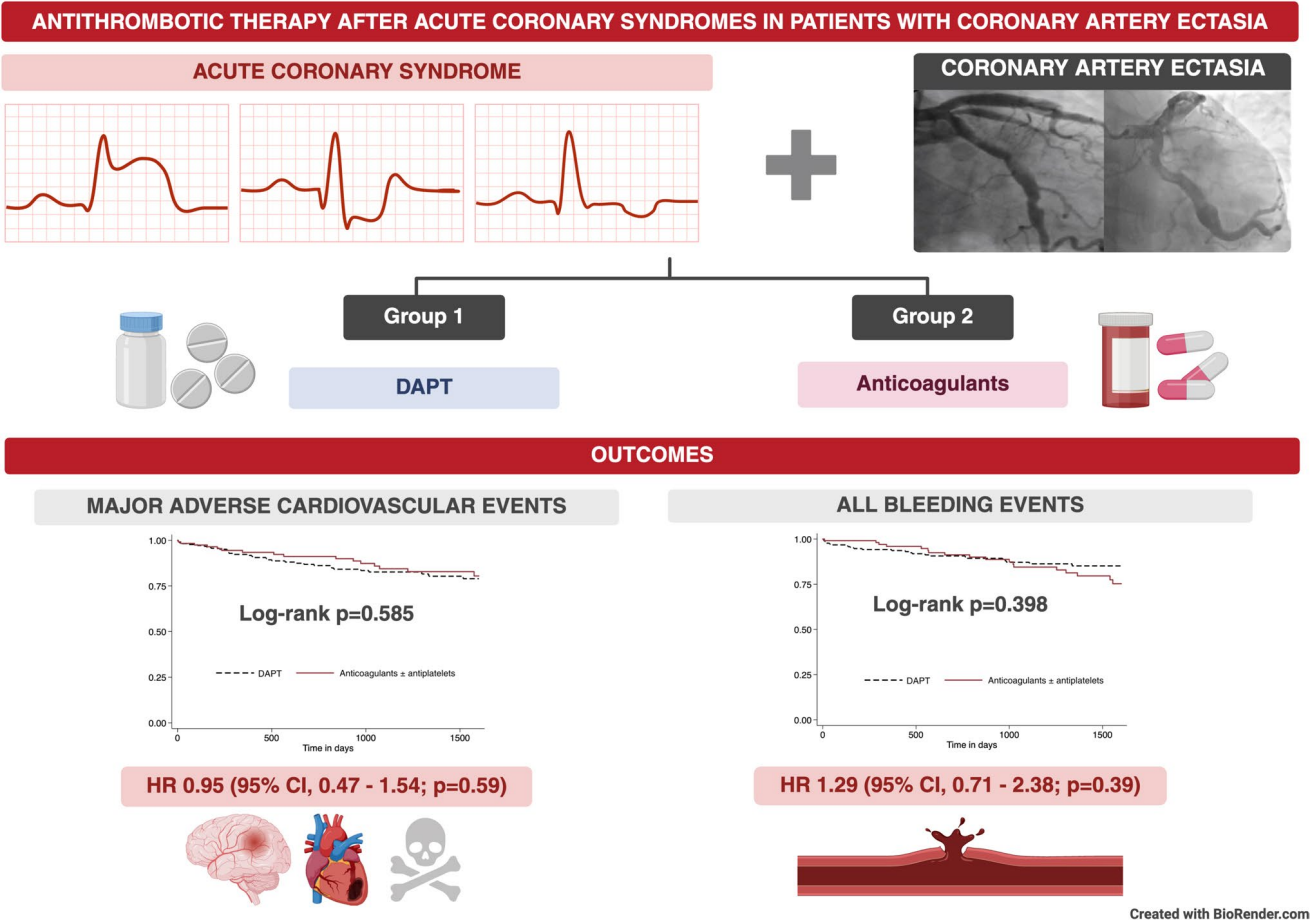

**Supplementary Information:**

The online version contains supplementary material available at 10.1007/s10557-025-07784-0.

## Introduction

Coronary artery ectasia (CAE) is a diffuse dilation of more than 1.5 times the most proximal segment that involves more than one-third of the vessel's length [1–3]. The exact cause of CAE is not fully understood, but it is likely multifactorial. About half of adult cases are linked to atherosclerosis, with shared mechanisms such as inflammation, lipid buildup, and tissue remodeling. Younger patients are more likely to develop CAE due to inflammatory or infectious causes, primarily Kawasaki disease or connective tissue disorders [4–6]. MicroRNAs, such as miR-133b, may be associated with its development [7, 8]. Additionally, CAE has been linked to elevated inflammatory markers and plasma-soluble adhesion molecules, which play significant roles in forming aneurysms [1–6, 9].

Regardless of the etiology, patients with CAE exhibit altered blood flow due to dilatation, leading to platelet activation and thrombus formation [9–14]. Patients with CAE have thus an increased risk for major adverse cardiovascular events (MACE); it’s found in the culprit artery in about 2% of individuals experiencing an acute coronary syndrome (ACS) [15]. As a result, various studies have proposed anticoagulation as the primary component of antithrombotic treatment [16, 18] . However, large-scale studies evaluating the role of anticoagulants compared to DAPT alone for preventing recurrent ischemic events in patients with ACS and CAE are needed.

The present study aimed to assess the effectiveness and safety of different antithrombotic strategies in patients with CAE after an ACS event.

## Methods

This study was a retrospective, observational, single-center study that included patients > 18 years old who presented with an ACS and were diagnosed with CAE in our center from 2012 to 2020. ACS was defined as the spectrum of myocardial infarction or unstable angina, in which a patient may present with chest pain or other clinical manifestations, with or without ECG changes, and with or without cTn elevation, as per ACC/AHA and ESC Guidelines [19–21]. Myocardial infarction, which includes STEMI and NSTEMI, was defined as evidence of myocardial injury, given the presence of elevated cTn of > 99th percentile of the upper reference limit with a rise and/or fall, with or without ST-segment elevation [19–21]. Unstable angina was defined as myocardial ischemia at rest or minimal exertion with the absence of myocardial injury, with angina lasting more than 20 min. [19–21]. For this study, the discharge diagnosis of ACS was retrospectively confirmed using the discharge records and verified according to the previously outlined definitions. CAE was defined as a dilation of > 1.5 times the most proximal segment of any coronary artery in a coronary angiography. Diagnosis and Markis classification were retrospectively assessed as reported on the Interventional Cardiology department’s electronic records, documented by an interventional cardiologist. Study investigators, who were either cardiology fellows or interventional cardiology fellows, then reviewed each angiography to confirm the presence of ectasia. The Markis Classification, which categorizes disease based on vessel involvement, was used to assess patients as follows: Type 1 corresponds to diffuse involvement in two or three vessels; Type 2 to diffuse involvement in one vessel and localized involvement in a second vessel; Type 3 to diffuse involvement in only one vessel; and Type 4 to localized ectasia in only one vessel [3]. High-grade ectasia was defined as either Markis 1 or Markis 2.

Patients were divided into two groups based on their medication regimen, which was previously chosen by the treating physician, based on preference, at discharge. Group 1 comprised patients receiving only dual antiplatelet therapy (DAPT), which could include combinations of aspirin with either clopidogrel, ticagrelor, or prasugrel. There were no patients on SAPT. Group 2 (Anticoagulants ± antiplatelets) included patients treated with anticoagulants such as Vitamin K Antagonists (VKAs) or Direct Oral Anticoagulants (DOACs), regardless of antiplatelet therapy. VKAs included warfarin or acenocoumarol, while DOACs included rivaroxaban, apixaban, and dabigatran. The dosage range for DOACs was as follows: rivaroxaban from 5 mg once daily (QD) to 20 mg QD, apixaban from 2.5 mg to 10 mg twice daily (BID), and dabigatran from 75 mg QD to 300 mg QD. Patients taking rivaroxaban 2.5 mg QD were excluded from the analysis, as this is not a therapeutic dose for anticoagulation. For patients on VKAs, the therapeutic time range (TTR) was calculated using the Rosendaal method [22] , which relies on multiple International Normalized Ratio (INR) measurements and their corresponding dates. Outpatient follow-up data were utilized. Patients in the anticoagulant group with a TTR of less than 60% were excluded from the analysis. However, an Intention to Treat analysis including patients with a TTR < 60% was made and can be found in the supplementary material. Given the retrospective nature of the study, treatment switches were permitted; however, for the purpose of analysis, patients were assigned to their initial treatment group. Data was collected from medical records by physicians.

The primary outcome was MACE, which was defined as the composite of all-cause mortality, reinfarction, and ischemic stroke. Secondary outcomes included individual MACE components and bleeding events, and the net adverse clinical events (NACE), which was defined as all MACE plus all bleeding. Bleeding events were subsequently categorized based on severity using the GUSTO bleeding scale. Follow-up for outcomes extended up to 1600 days, with data extracted from the chart registry. Loss to follow up was defined as follow up of less than 3 months. Outcome assessors, who were physician-researchers, were aware of treatment regimens but strictly reported diagnoses made by cardiologists as recorded in the registry.

We described and compared quantitative variables based on their distribution by treatment strategy (DAPT vs anticoagulants ± antiplatelets). Normally distributed variables, assessed by the Shapiro–Wilk test, were reported as means and standard deviations, and comparisons were made using Student’s t-test. Non-normally distributed variables were presented with medians and interquartile ranges, and Mann–Whitney’s test comparisons were performed. Categorical variables were described with frequencies and percentages and compared using the χ2 test.

To analyze the primary endpoint, we calculated the differences in the time to the event of composite MACE for each group using the Log-rank test and depicted the results using Kaplan–Meier curves. Similarly, the same analyses were done for secondary endpoints, which included each MACE component. Additionally, we used a univariate Cox regression model for the primary outcome to compare the effectiveness of the DAPT strategy with anticoagulants ± antiplatelets. We selected the statistically significant variables from these univariate models and included them in an adjusted model by age and sex. Statistical significance was determined at a p-value < 0.05, with a confidence interval of 95%. All analyses were performed on StataMP 14 software. To account for potential confounding by indication due to the non-randomized treatment assignment, a propensity score matched analysis was conducted. Propensity scores were estimated via logistic regression. The model used treatment regimen (DAPT vs. Anticoagulation) as the dependent variable and included sex, stenting, STEMI presentation, and high-grade ectasia as covariates. After analysis with a test for effect modification, and an adjusted analysis for confounding, these four variables were selected for propensity score matching. One-to-one (1:1) nearest neighbor matching was performed with R Studio to obtain the matched population. Analysis of the primary outcome, the secondary outcomes, and the composite of bleeding was then repeated on Stata after matching.

Patients or the public were not involved in the design, conduct, reporting, or dissemination plans of this research. This study used pre-existing data, and direct patient involvement was not deemed necessary.

## Results

**Fig. 1:**
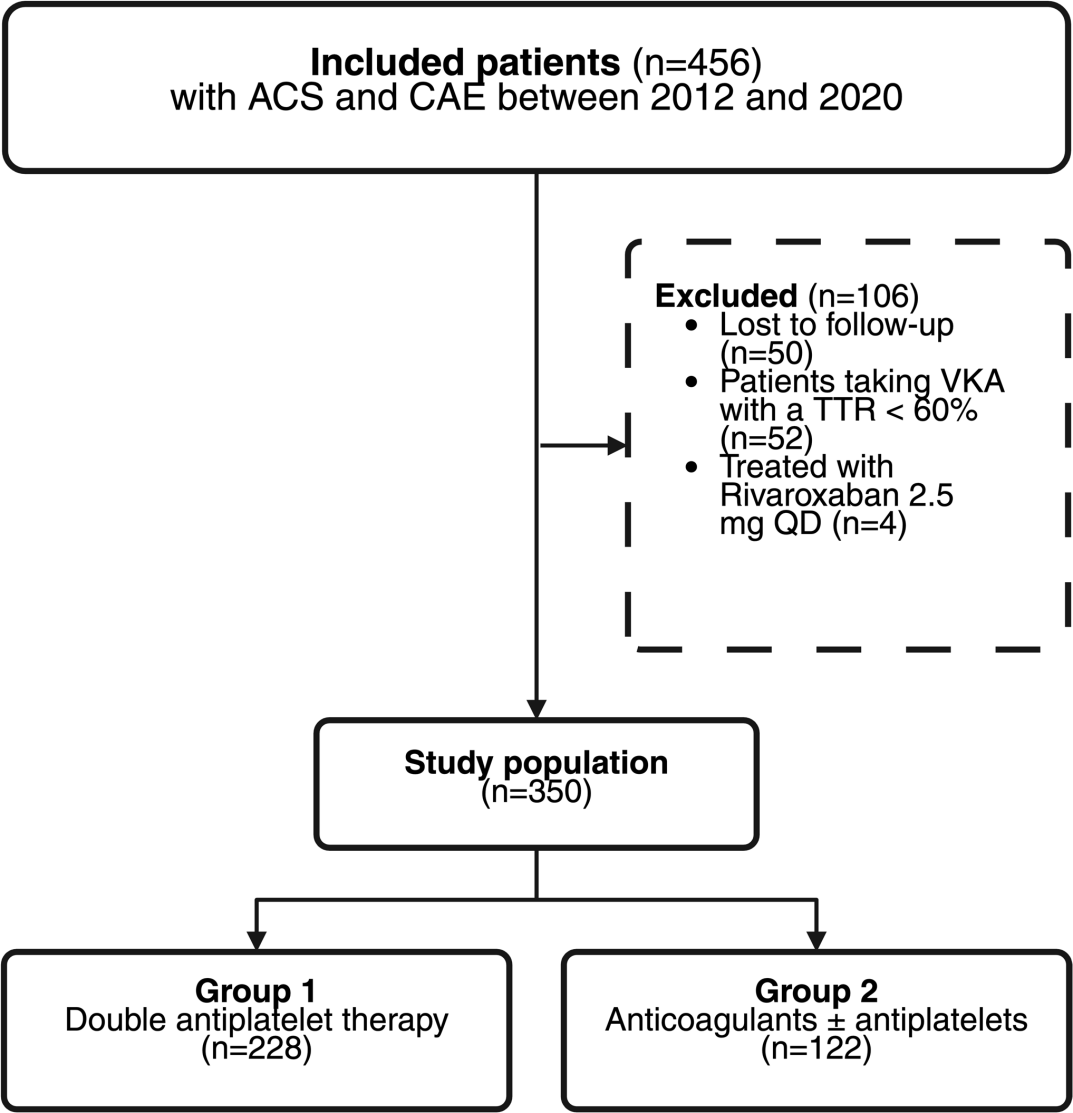
Flow chart of the study subjects

**Fig. 2:**
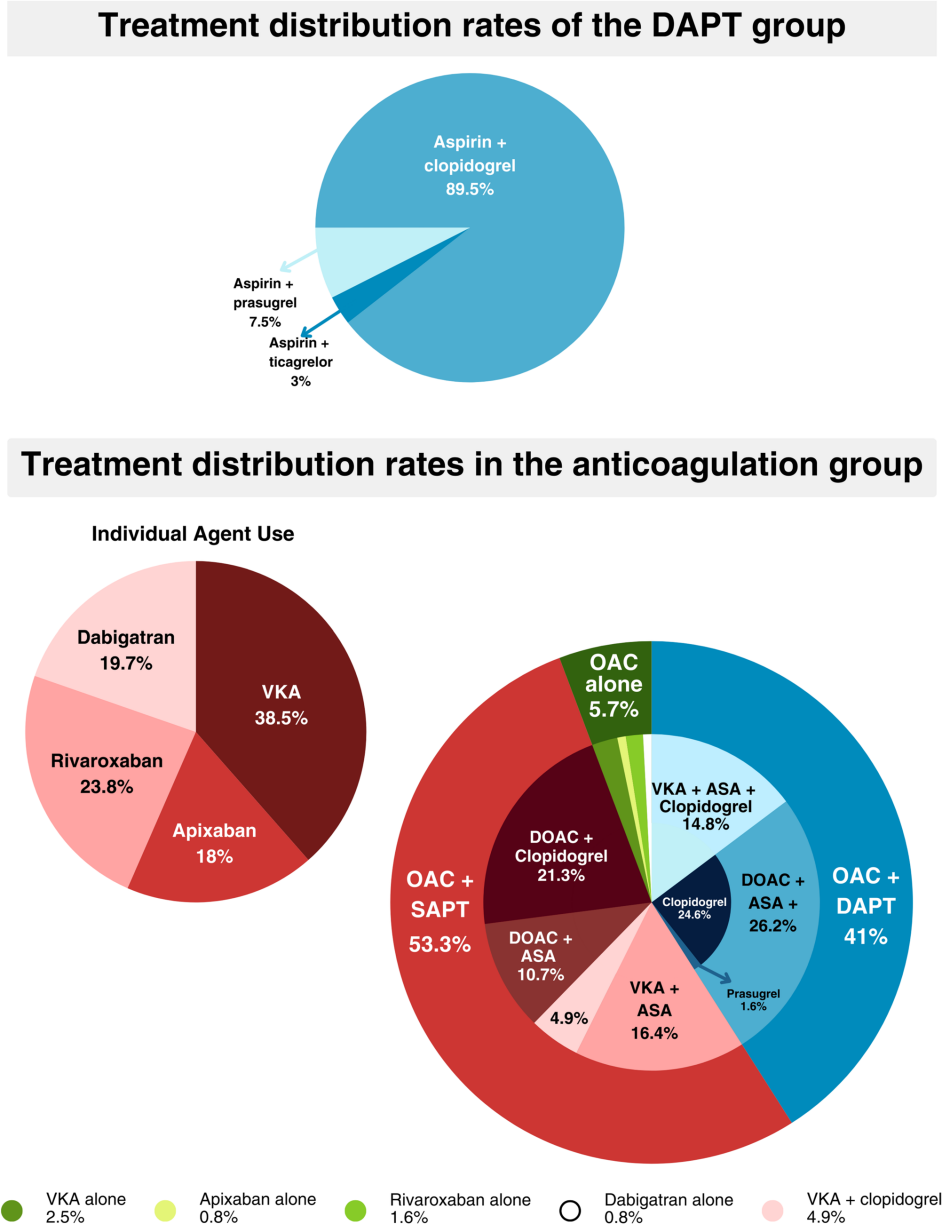
Treatment distribution

The disposition of patients is depicted in Fig. 1. Initially, 456 patients diagnosed with ACS and CAE between 2012 and 2020 were included. Of 109 patients on VKA, only 26 achieved a TTR of 60% or higher; the remaining 83 were excluded from the analysis. An intention-to-treat analysis, including patients whose TTR was below 60%, can be found in the supplementary material. Of patients taking rivaroxaban, four were on a non-therapeutic dosage of 2.5 mg QD and were excluded. Additionally, 19 patients were lost to follow-up. After excluding 106 patients, the final analysis comprised 350 patients, with 228 in the DAPT group and 122 in the anticoagulants ± antiplatelets group. Among the anticoagulation group, 41.0% were discharged with a triple therapy regimen, 53.3% with an anticoagulant plus SAPT, and 5.7% with an anticoagulant alone. A more detailed breakdown of treatment regimens is provided in Fig. 2.

Baseline characteristics are shown in Table 1. The average age was 58.7 years (± 11.4 years), and most patients were male (88.0%). The most frequent risk factors were a history of tobacco use (58.6%), hypertension (57.4%), and type 2 diabetes (21.2%). The latter was more prevalent in the DAPT group (25.6% vs 13.1%, *p = *0.007). The most common diagnosis at the time of admission was STEMI (64.0%). The DAPT group presented more frequently with NSTEMI (35.5% vs 21.3%, *p = *0.006), whereas the anticoagulants ± antiplatelets group presented more with STEMI (58.8% vs 73.8%, *p = *0.005). The anticoagulants ± antiplatelets showed higher Markis 1 rates (42.1% vs. 64.8%, *p = * < 0.001), while the DAPT group showed higher Markis 4 rates (21.9% vs 4.1%, *p = * < 0.001). The DAPT group was treated at a higher rate with stenting in the culprit artery (50.1% vs. 23.0%, *p = * < 0.001). No difference in renal or cardiac function at discharge was seen between both groups.

**Table 1:** Baseline characteristics

	Total Population (*n = *350)	DAPT (*n = *228)	Anticoagulants ± antiplatelets (*n = *122)	*p*-value
Demographics				
Male sex, *n (%)*	308 (88)	201 (88.2)	107 (88.2)	0.901
Age, *mean (SD)*	58.7 (± 11.4)	59.2 (± 11.9)	57.6 (± 10.3)	0.195
BMI,* median (IQR)*	28 (25.5—31.5)	27.6 (25—31.1)	28.7 (27.3—32.4)	0.004
Comorbidities, *n (%)*				
Hypertension	201 (57.4)	132 (57.9)	69 (56.6)	0.809
Type 2 diabetes	74 (21.2)	58 (25.6)	16 (13.1)	0.007
Tobacco use	204 (58.5)	130 (57.3)	74 (60.7)	0.540
CAE characteristics				
Markis Classification, *n (%)*				
Markis 1 and 2 (High grade)	227 (64.8)	125 (54.8)	102 (83.6)	< 0.001
Markis 3 and 4 (Low grade)	123 (35.2)	103 (45.2)	20 (16.4)	
Location, *n (%)*				
Left main coronary	81 (23.1)	43 (18.9)	38 (31.2)	0.009
Left descending artery-Diffuse LAD	230 (65.7) - 70 (20)	133 (58.3) - 32 (14)	97 (79.5) - 38 (31.2)	< 0.001 - < 0.001
Left circumflex artery-Diffuse LCX	189 (54) - 96 (27.4)	101 (44.3) - 44 (19.3)	88 (72.1) - 52 (42.6)	< 0.001 - < 0.001
Right coronary artery-Diffuse RCA	289 (82.6) - 159 (45.4)	181 (79.4) - 97 (42.5)	108 (88.5) - 62 (50.8)	0.032 - 0.138
Presentation, *n (%)*				
STEMI	224 (64)	134 (58.8)	90 (73.8)	0.005
NSTEMI	107 (30.6)	81 (35.5)	26 (21.3)	0.006
Unstable angina	19 (5.4)	13 (5.7)	6 (4.9)	0.758
Clinical Scores				
GRACE, *median (IQR)*	110.5 (87—133)	110 (87—132)	112 (91—139)	0.310
Killip-Kimball ≥ II, *n (%)*	81 (23.1)	55 (24.1)	26 (21.3)	0.552
TIMI, *n (%)*				
- 0–2	- 131 (37.4)	- 79 (34.7)	- 52 (42.6)	- 0.142
- 3–7	- 207 (59.1)	- 138 (60.5)	- 69 (56.6)	- 0.472
- ≥ 8	- 12 (3.4)	- 11 (4.8)	- 1 (0.8)	- 0.050
CRUSADE, *median (IQR)*	23 (16—32)	24 (16—34)	20.5 (17—29)	0.230
CHA_2_DS_2_-VASc ≥ 2*, n (%)*	211 (60.3)	144 (63.2)	67 (54.9)	0.133
HAS-BLED ≥ 2*, n (%)*	173 (49.4)	122 (53.5)	51 (41.8)	0.037
Treatment, *n (%)*				
Thromboaspiration	36 (10.3)	17 (7.5)	19 (15.6)	0.018
GpIIa/IIIb inhibitor	109 (30.2)	60 (26.4)	49 (40.2)	0.017
Stenting				
Stent in the culprit artery	143 (41)	115 (50.1)	28 (23)	< 0.001
Stent in other arteries	50 (14.3)	42 (18.5)	8 (6.6)	0.002
Type				
- Bare metal	- 68 (19.4)	- 54 (23.7)	- 14 (11.5)	- 0.006
- Drug-eluting	- 107 (30.6)	- 88 (38.6)	- 22 (17.5)	- < 0.001
TIMI after stenting ≥ 2*, n (%)*	290 (82.9)	199 (87.3)	91 (74.6)	0.003
TMP ≥ 2*, n (%)*	301 (86)	205 (89.9)	96 (78.7)	0.004
Complications at discharge				
Heart failure, *n (%)*	91 (27.4)	54 (25.6)	37 (30.3)	0.513
LVEF, *median (IQR)*	52 (42.5—59)	52.5 (42—60)	51.5 (43—58)	0.643
CKD, *n (%)*	38 (10.9)	26 (11.5)	12 (9.8)	0.644
GFR, *median (IQR)*	88 (74—101)	88 (74—101)	88.5 (75—101)	0.602
Follow up				
Follow-up in days, *median (IQR)*	1040 (329—1684)	1027 (318—1615)	1111 (335—1799)	0.498
Time on treatment in days, *median (IQR)*	553 (102—1404)	401 (50—945)	1205.5 (337—1945)	< 0.001
Lost to follow-up, n (%)	87 (24.9)	63 (27.6)	24 (19.7)	0.101

*CAE* coronary artery ectasia, *CKD* chronic kidney disease, *DAPT* double antiplatelet therapy, *GFR* glomerular filtration rate, *IQR* interquartile range, *LCX* left circumflex artery, *LAD* left descending artery, *LVEF* left ventricular ejection fraction, *NSTEMI* non-ST elevation myocardial infarction, *RCA* right coronary artery, *SD* standard deviation, *STEMI* ST-elevation myocardial infarction

Median follow-up was 1040 days, with no statistical difference between groups (1027 vs 1111, *p = *0.49). The median time on treatment was 553 days, with the anticoagulation group showing a longer time on treatment (401 vs 1206 days, *p = * < 0.001). The median time for treatment for each antithrombotic agent was calculated in 59% of the population and can be found in our supplementary material. The rate of loss to follow-up was 24.9%.

As for the primary endpoint, we found no statistically significant difference in the risk of MACE throughout follow-up for those patients receiving anticoagulants ± antiplatelets (HR 0.95; 95% CI, 0.47–1.54; *p = *0.59) compared with those receiving DAPT. The DAPT group showed 33 events, and the anticoagulants ± antiplatelets group showed 16 events (14.5% vs 13.0%, *p = *0.49).

Regarding the secondary endpoints, each of the individual components of MACE did not exhibit differences between groups either. Table 2 shows the primary and secondary endpoints. There were 11 all-cause mortality events, 40 reinfarction events, and four stroke events.

**Table 2:** Outcomes

	DAPT (*n = *228)	Anticoagulants ± antiplatelets (*n = *122)	*p*-value	HR (95%CI)	*p* value^2^
Efficacy, composite *n* (%)					
Composite of all-cause mortality, reinfarction, stroke	33 (14.5)	16 (13.1)	0.727	0.947 (0.47—1.54)	0.585
Efficacy, components of the composite outcome, *n* (%)					
All-cause mortality	10 (4.4)	1 (0.8)	0.068	0.18 (0.02—1.41)	0.103
Reinfarction	26 (11.4)	14 (11.6)	0.963	0.31 (0.49—1.8)	0.851
Stroke	2 (0.9)	2 (1.6)	0.541	1.73 (0.24—12.3)	0.586
Bleeding, composite outcome					
Bleeding	25 (11)	18 (14.8)	0.303	1.29 (0.71—2.38)	0.399
Bleeding, components of the composite outcome, *n* (%)					
GUSTO mild	9 (3.9)	16 (13.1)	0.002	3.19 (1.41—7.23)	0.005
GUSTO moderate	15 (6.6)	2 (1.6)	0.041	0.24 (0.06—1.06)	0.059
GUSTO severe or life-threatening	1 (0.44)	0 (0)	0.464	-	-
Net adverse clinical events (NACE), composite outcome					
Composite of death, reinfarction, stroke, and bleeding, *n* (%)	52 (22.8)	30 (24.6)	0.707	1.02 (0.65—1.6)	0.928

*CI* confidence interval, *DAPT* double antiplatelet therapy, *HR* hazard ratio

As for safety, bleeding as a composite outcome was also similar among groups, with no statistically significant differences (HR 1.29, 95% CI 0.71–2.38, *p = *0.39) with a total of 43 events (*n = *25, 11.0% vs *n = *18, 14.8%; *p = *0.303). However, 16 (13.1%) GUSTO mild bleeding events were observed in the anticoagulants ± antiplatelets group, which was statistically higher than the DAPT group, which presented with 9 (3.9%) events. Logistic regression showed a hazard ratio of 3.19 (95% CI, 1.41—7.23; *p = *0.005). Otherwise, moderate and severe GUSTO bleeding events did not differ.

**Fig. 3:**
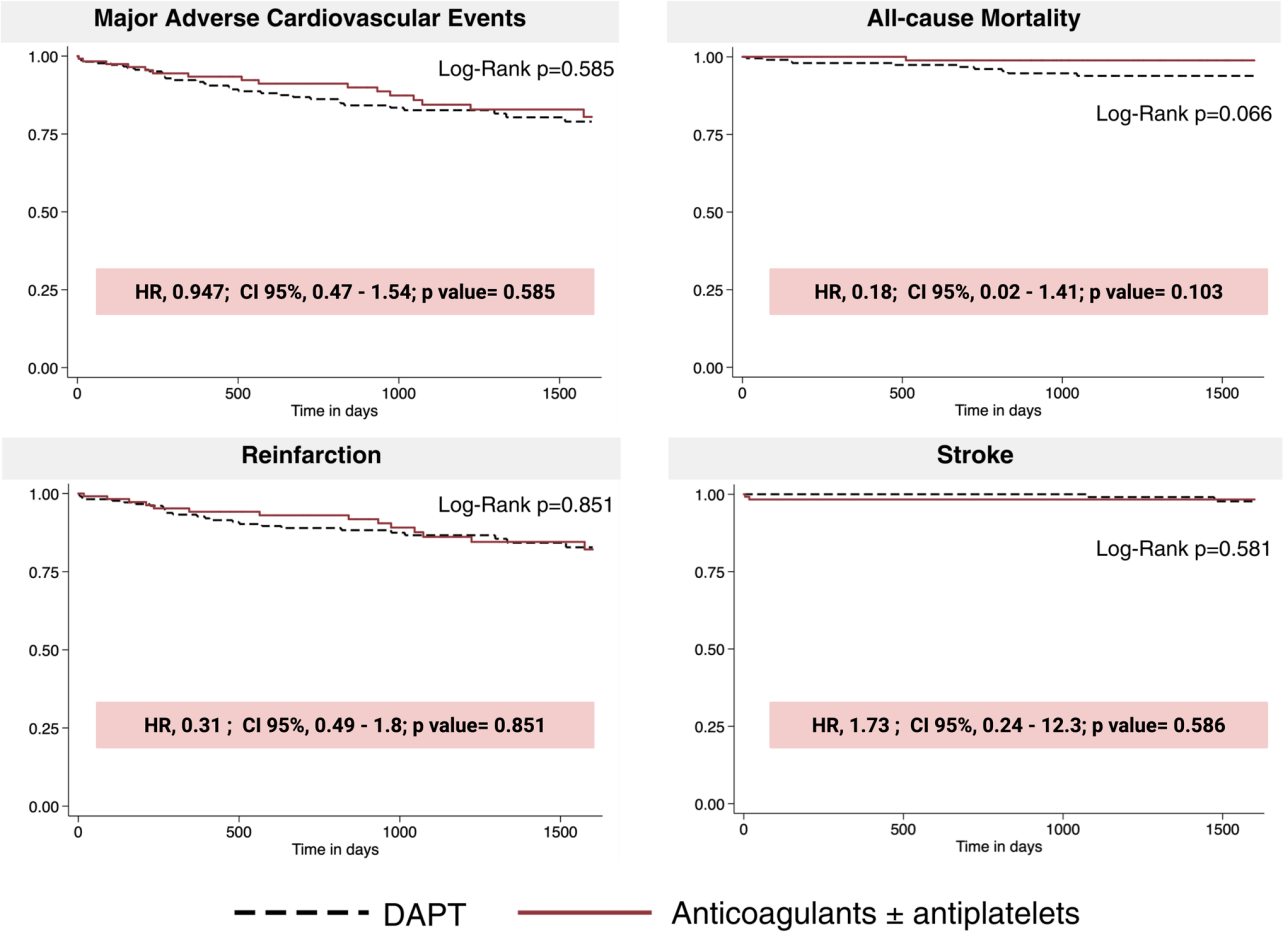
MACE and its components

NACE did not display a difference between groups either (22.8% vs 24.6%, *p = *0.71). Figure 3 depicts the occurrences of MACE, highlighting the differences between the two groups. Table 2 presents the occurrences of MACE, bleeding, and NACE events and compares the groups.

On univariate analysis, diffuse ectasia of the LAD (HR = 2.14, *p = *0.015) and LVEF at discharge < 50% (HR = 1.88, *p = *0.029) were associated with the main outcome. After multivariate analysis adjusted for age and sex, both associations with the main outcomepersisted, with diffuse LAD involvement associated with a 2.28 HR (95% CI, 1.2—4.2; *p = *0.01), and LVEF at discharge < 50% associated with a 1.89 HR (95% CI, 1.1—3.8; *p = *0.03). Univariate and multivariate analyses can be seen in Table 3.

**Table 3:** Univariate and adjusted multivariate analysis adjusted by group and diabetes

	Univariate analysis		Multivariate adjusted analysis by age, sex, and anticoagulation treatment	
	HR (95% CI)	*p*-value	HR (95% CI)	*p*-value
*Demographic characteristics*				
Male sex	1.04 (0.44—2.44)	0.931	––	––
Age	1.02 (0.99—1.05)	0.110	––	––
Hypertension	0.93 (0.53—1.63)	0.788	0.86 (0.48—1.54)	0.611
Type 2 diabetes	1.22 (0.63—2.33)	0.557	1.23 (0.64—2.4)	0.534
Tobacco Use	0.96 (0.54—1.69)	0.881	0.97 (0.55—1.73)	0.923
BMI ≥ 30	0.81 (0.45—1.48)	0.497	0.94 (0.50—1.78)	0.860
Markis ≤ 2 (High grade)	1.40 (0.75—2.6)	0.287	1.56 (0.82—2.97)	0.179
*Ectasia in culprit artery location:*				
Left main coronary	1.58 (0.85—2.94)	0.059	1.65 (0.88—3.1)	0.117
Left descending - Diffuse LAD	1.3 (0.7—2.42) - **2.14 (1.16—3.95)**	0.407 - **0.015**	1.4 (0.74—2.63) - **2.28 (1.22—4.24)**	0.296 - **0.010**
Circumflex - Diffuse LCX	1.58 (0.88—2.83) - 1.61 (0.88—2.93)	0.122 - 0.122	1.73 (0.94—3.17) - 1.82 (0.96—3.44)	0.076 - 0.065
Right coronary - Diffuse RCA	1.42 (0.54—2.44) - 1.69 (0.96—2.98)	0.730 - 0.067	1.19 (0.55—2.55) - 1.72 (0.97—3.03)	0.662 - 0.062
*Clinical presentation and treatment*				
STEMI	1.06 (0.59—1.88)	0.854	1.17 (0.64—2.14)	0.605
Stenting	1.07 (0.6—1.91)	0.821	1.04 (0.55 1.94)	0.908
TIMI after stenting ≥ 2	0.60 (0.32—1.14)	0.118	0.6 (0.31—1.15)	0.122
TMP ≥ 2	0.78 (0.38—1.6)	0.497	0.74 (0.36—1.55)	0.427
LVEF at discharge below 50%	**1.93 (1.1—3.38)**	**0.021**	**1.89 (1.08—3.33)**	**0.026**
Anticoagulation treatment	0.85 (0.47—1.54)	0.585	––	––

*ACS* acute coronary syndrome, *BMI* body mass index, *CI* confidence interval, *HR* hazard ratio, *LCX* left circumflex artery, *LAD* left descending artery, *LVEF* left ventricular ejection fraction, *RCA* right coronary artery, *STEMI* ST-elevation myocardial infarction, *TMP* TIMI myocardial perfusion

Propensity Score Matching analysis showed 0.0% bias on the matched population by the variables: stenting, STEMI presentation, high-grade ectasia, and male sex. Repeat analysis of the primary outcome in the matched population showed no statistically significant difference between groups (HR 0.65, 95% CI 0.34—1.26, *p = *0.204), with 21 (17.2%) events in the DAPT group and 15 (12.3%) events in the group taking anticoagulants ± antiplatelets (*p = *0.279). No significant differences were found in each of the individual components of MACE, or on the composite of bleeding events. This analysis can be found on the supplementary material “Propensity score matching analysis”.

## Discussion

In this real-world observational study, we did not find evidence to support the use of anticoagulants in addition to antiplatelets in patients with CAE after an ACS. Our series represents the largest dataset to date comparing DAPT versus anticoagulation in this population with such prolonged follow-up. In our cohort, neither composite MACE, its individual components, nor composite bleeding events showed a statistically significant difference between both groups. These results persisted after propensity score matching analysis.

Our results contrast with those previously published. Doi et al. studied outcomes in 8 patients after acute MI with CAE taking warfarin whose TTR was above 60% compared with 43 patients whose TTR was below 60% or who were not taking warfarin [17]. Patients taking warfarin presented a statistically significant lower prevalence of reinfarction and cardiovascular death. However, a group taking DAPT was not analyzed in Doi’s study. Pranata et al. analyzed a series of cases of patients taking anticoagulation therapy versus single antiplatelet therapy (SAPT) or DAPT. Thirteen cases were studied where those taking optimal anticoagulation had a lower recurrence rate, with a mean follow-up of 8.4 months. Only 2 out of the 13 were taking DOACs [18]. Another 3 cases were described by Yan et al. where patients taking a DOAC had no ACS recurrence or any other MACE with a maximum follow-up of 12 months [23]. Our study had a median follow-up of about 32 months, contrasting with the studies previously mentioned. Additionally, Amirpour et al. (2024) published a systematic review including 457 patients, incorporating the studies mentioned above, which compared antiplatelet and anticoagulant regimens in relation to major adverse cardiovascular events (MACE). They concluded that combining antiplatelet therapy with anticoagulants may reduce MACE recurrence [24]. However, it must be pointed out that most of the included studies were case reports, some with short follow-up periods. Only one randomized controlled trial was identified, but at the time, it had only published its rationale, and its outcomes were not included. This RCT, published by Araiza-Garaygordobil (2024), has since reported results, observing no statistical difference in MACE between patients treated with DAPT (aspirin 100 mg daily plus clopidogrel 75 mg daily) and those treated with SAPT + OAC (Rivaroxaban 15 mg daily plus clopidogrel 75 mg daily) [25]. More recently, Azarboo et al. (2025) conducted a systematic review and network meta-analysis that included 1,106 patients divided into four treatment groups: dual antiplatelet therapy (DAPT), anticoagulants, aspirin alone, and no treatment. Their findings demonstrated that DAPT was the most effective strategy for preventing MACE in patients with coronary artery ectasia (CAE), followed by aspirin alone, anticoagulants, and no treatment. These results not only align with our findings—supporting the non-superiority of anticoagulants over DAPT—but also suggest that DAPT may lead to improved clinical outcomes and fewer adverse events [26].

Importantly, anticoagulant treatment has evolved substantially in the last few decades, with DOACs emerging as valuable and effective therapies [27]. In this particular clinical scenario, DOAC effectiveness in ACS has only been reported as clinical cases, lacking sufficient evidence to either rule in or rule out its use as treatment. In our study, DOAC effectiveness was analyzed along with VKAs as a whole entity.

A second finding in our population was a higher incidence of GUSTO mild bleeding events with anticoagulation therapy, while antiplatelet therapy was associated with higher GUSTO moderate bleeding events. The latter might be explained by a higher percentage of patients in the DAPT group with a HAS-BLED score ≥ 2. However, composite bleeding events and net adverse clinical events did not differ among groups. Our findings suggest that both groups of patients have similar outcomes. Still, patients treated with anticoagulants may have an increased risk of mild bleeding, and the relatively small sample size may obscure the risk of major bleeding. In patients after ACS without CAE, DOAC users have been found to have a lower recurrence of MACE at the expense of increasing major bleeding in a dose-dependent manner [28, 29].

A third key finding in our study was the difference in clinical presentation and treatment between groups. In our population, the anticoagulants ± antiplatelets group presented more frequently with STEMI. They were also less likely to have been treated with a stent and more likely to have been treated with thromboaspiration and/or a GpIIa/IIIb inhibitor, probably due to the severity of the ectasia. The prevalence of TIMI scores after intervention and TMP scores above 2 was higher in the DAPT group. A likely explanation is the variation in clinical presentation, severity of ectasia, and treatment among groups. Treatment strategies may influence determining if anticoagulation has a role in MACE prevention, as patients who have undergone thromboaspiration rather than stenting may have a stronger indication for anticoagulation. Further studies on additional factors and how they influence treatment indications must be conducted, including stenting, number of stents used, thromboaspiration, and GpIIa/IIIb inhibitor use.

Moreover, CAE encompasses several subgroups of patients with distinct characteristics, including varying numbers of affected arteries and the extent of involvement in each artery. The anticoagulants ± antiplatelets group had lower Markis scores in our population, indicating more diffuse disease. In our multivariate analysis, diffuse presentations of all three arteries were associated with the primary outcome. A previous study by Gunasekaran et al. categorized Markis 1 and 2 as high-grade ectasia and Markis 3 and 4 as low-grade ectasia [16]. High-grade disease had a significantly higher rate of ACS, and antithrombotic therapy was associated with a lower rate [16]. Additionally, certain ectasia locations may be associated with a higher risk of MACE and may warrant anticoagulation therapy; as in our multivariate analysis, LAD location was significantly associated with the main outcome. All the factors previously stated in this paragraph lead us to question if we should define the use of anticoagulation depending not only on the Markis classification but also on the severity and location of the ectasia itself.

Our findings should be interpreted considering certain limitations. A first limitation was that, to broaden our study population, the DAPT group and the anticoagulants ± antiplatelets group included different treatment combinations and different doses of anticoagulants. No specific drug combination was studied. We propose further analysis with treatment subgroups or standardized treatment.

Our study lacked randomization in grouping due to its nature, so the baseline characteristics differed. In addition to those previously mentioned, the DAPT group was more likely to receive a stent, while the anticoagulants ± antiplatelets group had greater use of GpIIb/IIIa inhibitors. This suggests the possibility of different causal mechanisms in each group, with a predominance of classic atheromatous disease in the DAPT group and in situ thrombus formation in the anticoagulants ± antiplatelets group. On the other hand, although both groups have coronary ectasia, it’s possible that some patients didn’t receive a stent due to a more severe disease phenotype characterized by larger arterial diameters. On several occasions, this hindered stent placement and limited treatment to thrombectomy and GpIIb/IIIa inhibitors. Even with propensity score matching to address this, the evidence remains less definitive than that from an RCT.

It’s essential to consider these limitations since, while the clinical outcomes were not significant, having a different causal mechanism for acute coronary syndrome or different spectra of the same disease may influence patient prognosis, regardless of the treatment received. The evidence remains controversial; traditionally, the Markis classification associates worse prognosis with increased vessel involvement. However, recent studies suggest that vessel size does not impact MACE occurrence [30].

Despite the limitations previously mentioned, this study lays a foundation for better determining a gold standard treatment for CAE. Our analysis suggests that DAPT therapy may have the same effectiveness as anticoagulation in this population. If this is true, other factors support DAPT, as it carries a lower financial burden than DOACs. Additionally, antiplatelet therapy has fewer interactions than VKAs, and there’s no need to measure INR to ensure patients stay within the therapeutic range.

Currently, more randomized controlled trials are comparing standard DAPT versus anticoagulation in CAE patients with a primary endpoint of MACE [31, 32]. We await results to guide therapy for CAE.

## Conclusion

Our analysis explored the effectiveness and bleeding risk associated with DAPT and anticoagulation in patients presenting with ACS and CAE. While our findings suggest that DAPT may offer a favorable balance of MACE prevention and lower bleeding risk compared to anticoagulation in this heterogeneous patient population, a direct comparison is limited by the diverse characteristics of the groups and the varied medical regimens employed.

These results prompt a critical re-evaluation of current medical practices regarding the routine prescription of anticoagulants in all ACS patients with CAE. We do not discount the potential role of anticoagulants in managing coronary ectasia, particularly in specific subgroups. However, further research is essential to delineate the optimal candidates for anticoagulation therapy in ACS with CAE. Future studies should focus on ectasia severity, clinical presentation, and specific treatment strategies to provide more tailored and evidence-based recommendations.

## Supplementary Information

Below is the link to the electronic supplementary material.

Supplementary file1 (DOCX 344 KB)

Supplementary file2 (DOCX 226 KB)

Supplementary file3 (DOCX 308 KB)

Supplementary file4 (DTA 211 KB)

## Data Availability

The data that support the findings of this study are available from the corresponding author, FSJ, upon reasonable request.

## Code Availability

The code used in StataMP that support the findings of this study are available from the corresponding author, FSJ, upon reasonable request.
