# Supplementary material for: Anticoagulant versus Antiplatelet Therapy After Acute Coronary Syndromes in Patients with Coronary Artery Ectasia: A Retrospective Cohort Study
Source: Cardiovasc Drugs Ther. 2025 Sep 24;40(3):1021–33. doi: 10.1007/s10557-025-07784-0 (PMC13171775; doi:10.1007/s10557-025-07784-0)
Supplement: Supplementary file 1 — Supplementary file1 (DOCX 344 KB) [file 10557_2025_7784_MOESM1_ESM.docx]

# Supplementary material - Treatment regimens and duration

# Table 4. Treatment distribution

|  | **Total Population**  **(n=350)** | **DAPT**  **(n=228)** | **Anticoagulants ± antiplatelets (n=122)** |
| --- | --- | --- | --- |
| **Overall Medication Use, n (%)** | | | |
| *Antiplatelets* | | | |
| Aspirin | 309 (88.3) | 228 (100) | 83 (68) |
| Clopidogrel | 284 (81.1) | 204 (89.5) | 80 (65.7) |
| Prasugrel | 19 (5.4) | 17 (7.5) | 2 (1.6) |
| Ticagrelor | 7 (2) | 7 (3.1) | 0 (0) |
| *Anticoagulants* | | | |
| Acenocoumarol |  | | 47 (38.5) |
| Apixaban   - 5 mg BID - 10 mg BID |  |  | 22 (18)   - 7 (5.7) - 15 (12.3) |
| Rivaroxaban   - 5 mg QD - 15 mg QD - 20 mg QD |  |  | 29 (23.8)   - 6 (4.9) - 8 (6.6) - 15 (12.3) |
| Dabigatran   - 110 mg QD - 220 mg QD - 300 mg QD |  |  | 24 (19.7)   - 1 (0.8) - 2 (2.5) - 21 (16.4) |
| **Treatment combinations, n(%)** | | | |
| *DAPT combinations* | | | |
| Aspirin + clopidogrel |  | 204 (89.5) |  |
| Aspirin + prasugrel |  | 17 (7.5) |  |
| Aspirin + ticagrelor |  | 7 (3.1) |  |
| *Patients in anticoagulants* | | | |
| Acenocoumarol alone |  | | 3 (2.5) |
| Apixaban alone |  |  | 1 (0.8) |
| Rivaroxaban alone |  |  | 2 (1.6) |
| Dabigatran alone |  |  | 1 (0.8) |
| Acenocoumarol + aspirin |  |  | 20 (16.4) |
| Acenocoumarol + clopidogrel |  |  | 6 (4.9) |
| Acenocoumarol + ticagrelor |  |  | 0 (0) |
| Acenocoumarol + prasugrel |  |  | 0 (0) |
| DOAC + aspirin |  |  | 13 (11.5) |
| DOAC + clopidogrel |  |  | 26 (21.3) |
| DOAC + ticagrelor |  |  | 0 (0) |
| DOAC + prasugrel |  |  | 0 (0) |
| *Patients in triple therapy* | | | |
| Any triple treatment (OAC + DAPT) |  | | 50 (41) |
| - VKA + DAPT |  |  | 18 (14.7) |
| - DOAC + DAPT |  |  | 32 (26.2) |

**Table 5.** Duration of treatment in days analysis with 59.1% of the population.

|  | **Total Population**  **(n=207)** | **DAPT**  **(n=98)** | **Anticoagulants ± antiplatelets (n=109)** |
| --- | --- | --- | --- |
| DAPT | 493.5 (109 - 926) | 596 (147 - 1026) | 141 (70 - 340) |
| Any antiplatelets | 857 (284 - 1733) | 857 (195 - 904) | 861.5 (333 - 1555) |
| - Aspirin | 883 (195 - 1845) | 823 (195 - 1845) | 1021.5 (169 - 1781.5) |
| - Clopidogrel | 506 (181 - 934.5) | 596 (142 - 1038) | 445 (227 - 831) |
| - Ticagrelor | 1148 (753 - 1543) | 1148 (753 - 1543) |  |
| - Prasugrel | 436 (81 - 813) | 436 (81 - 813) |  |
| OAC |  | | 1111 (341 - 1859) |
| - VKA |  |  | 1645.5 (1033 - 2141) |
| - Rivaroxaban |  |  | 272 (175 - 1104) |
| - Apixaban |  |  | 980 (461 - 1581) |
| - Dabigatran |  |  | 1122.5 (341 - 1909) |
| Triple therapy |  |  | 141 (70 - 638) |

#

# Supplementary Figure 1. Median treatment duration times by group

#
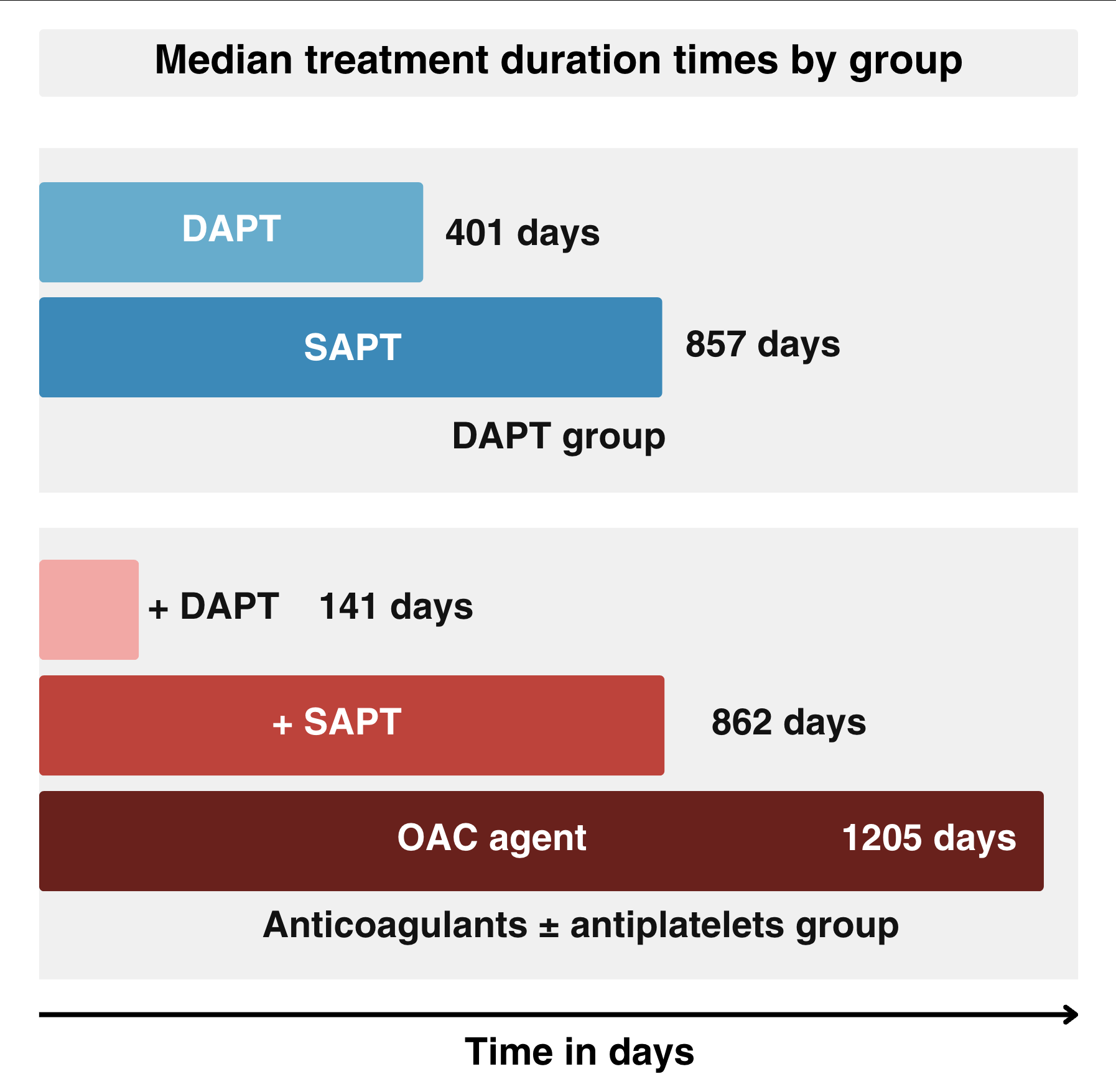


#

#

# Supplementary Figure 2. Median antiplatelet agent duration times by group boxplot.

#
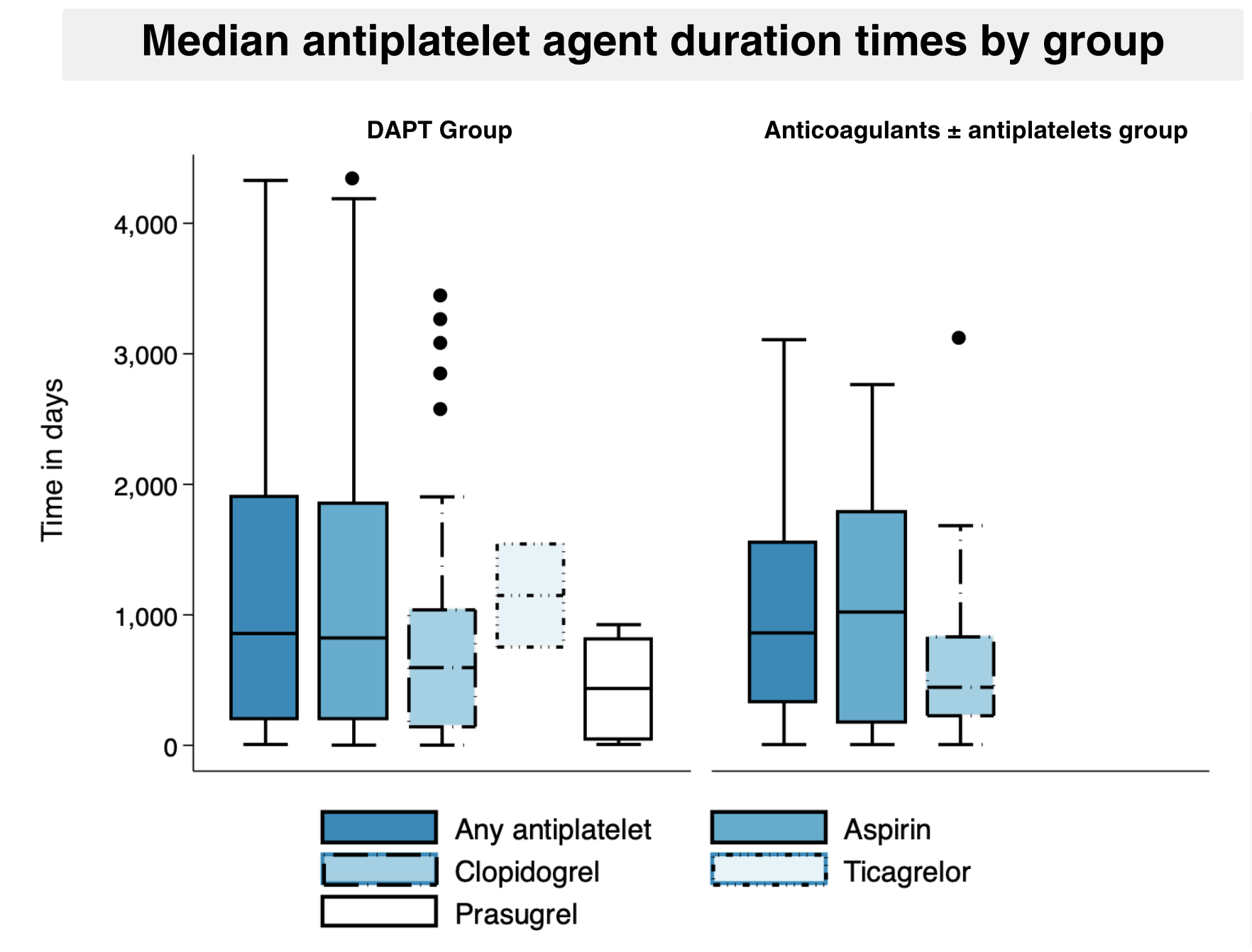


#

# Supplementary Figure 3. Median anticoagulant agent duration times boxplot.


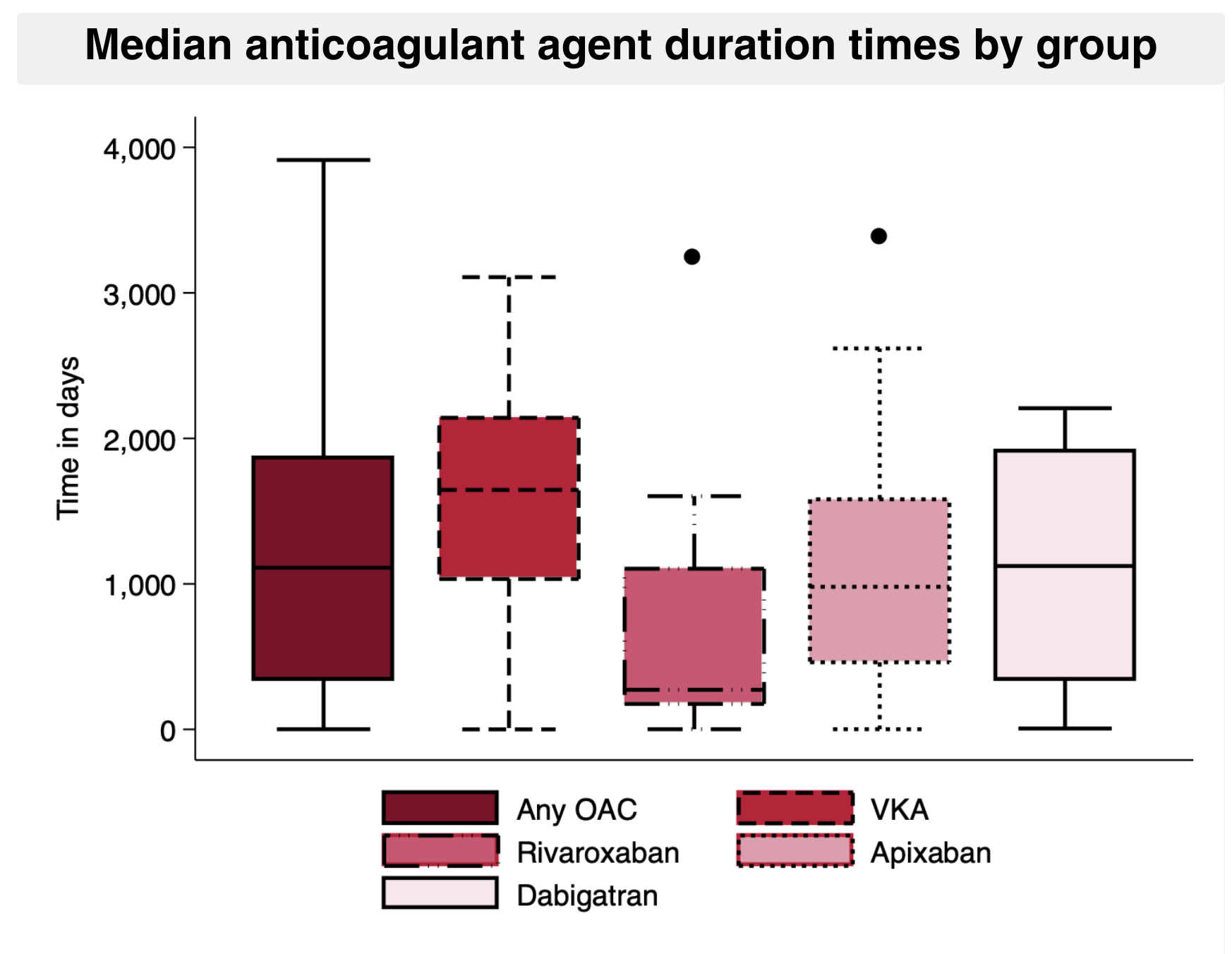


# 
