# Supplementary material for: Anticoagulant versus Antiplatelet Therapy After Acute Coronary Syndromes in Patients with Coronary Artery Ectasia: A Retrospective Cohort Study
Source: Cardiovasc Drugs Ther. 2025 Sep 24;40(3):1021–33. doi: 10.1007/s10557-025-07784-0 (PMC13171775; doi:10.1007/s10557-025-07784-0)
Supplement: Supplementary file 2 — Supplementary file2 (DOCX 226 KB) [file 10557_2025_7784_MOESM2_ESM.docx]

# Supplementary Material - Intention to Treat Analysis

**Supplementary table 1.** Baseline characteristics

|  | **Total Population**  **(n=403)** | **DAPT**  **(n=228)** | **Anticoagulants ± antiplatelets (n=175)** | **p-value** |
| --- | --- | --- | --- | --- |
| **Demographics** | | | | |
| Male sex, *n ( %)* | 358 (88.8) | 201 (88.2) | 157 (89.7) | 0.623 |
| Age, *mean (SD)* | 57.9 (±11.5) | 59.2 (±11.8) | 56.1 (±10.7) | 0.006 |
| Weight (kg), *median (IQR)* | 80 (71-90) | 79 (70-88) | 84 (75-94) | 0.001 |
| Height (cm), *median (IQR)* | 170 (164-173) | 168 (164-172) | 170 (164-175) | 0.094 |
| BMI, *median (IQR)* | 28 (25.5-31.7) | 27.6 (25-31.1) | 28.7 (26.4-32.7) | 0.004 |
| **Comorbidities, *n (%)*** | | | | |
| Hypertension | 222 (55.1) | 132 (57.9) | 90 (51.4) | 0.196 |
| Type 2 diabetes | 83 (20.7) | 58 (25.5) | 25 (14.3) | 0.006 |
| Tobacco use | 236 (58.7) | 130 (57.3) | 106 (60.6) | 0.505 |
| **CAE characteristics** | | | | |
| Markis Classification, *n (%)* | | | | |
| Markis 1 and 2 (High-grade) | 269 (66.7) | 125 (54.8) | 144 (82.3) | <0.001 |
| Markis 3 and 4 (Low grade) | 134 (33.3) | 103 (45.2) | 31 (17.7) | <0.001 |
| Location, *n (%)* | | | | |
| Left main coronary | 94 (23.3) | 43 (18.9) | 39 (31) | 0.033 |
| Left descending artery | 271 (67.3) | 133 (58.3) | 138 (78.9) | <0.001 |
| Left circumflex artery | 225 (55.8) | 101 (44.3) | 124 (70.9) | <0.001 |
| Right coronary artery | 335 (83.1) | 181 (79.4) | 154 (88) | 0.060 |
| **Presentation, *n (%)*** | | | | |
| STEMI | 261 (64.8) | 134 (58.8) | 127 (72.6) | 0.016 |
| NSTEMI | 122 (30.3) | 81 (35.5) | 41 (23.4) |  |
| Unstable angina | 20 (5) | 13 (5.7) | 7 (4) |  |
| **Follow up, *median (IQR)*** | | | | |
| Maximum follow up in days | 1046 (329-1600) | 1027.5 (318-1600) | 1065 (329-1600) | 0.294 |
| Time on treatment in days | 593 (123 - 1554) | 430 (54 - 1042) | 1008 (270 - 1600) | <0.001 |

**Supplementary table 2.** Treatment distribution

|  | **Total Population**  **(n=403)** | **DAPT**  **(n=228)** | **Anticoagulants ± antiplatelets (n=175)** | **p-value** |
| --- | --- | --- | --- | --- |
| Antiplatelets | | | | |
| Aspirin | 356 (88.3) | 228 (100) | 130 (74.3) | <0.001 |
| Clopidogrel | 317 (78.6) | 204 (89.4) | 113 (64.6) | <0.001 |
| Prasugrel | 19 (4.7) | 17 (7.5) | 2 (1.1) | 0.003 |
| Ticagrelor | 7 (1.7) | 7 (3.1) | 0 (0) | 0.019 |
| Anticoagulants | | | | |
| Acenocoumarol | 100 (24.8) |  | 100 (57.1) | <0.001 |
| Apixaban | 22 (5.4) |  | 22 (12.6) | <0.001 |
| Rivaroxaban | 29 (7.2) |  | 29 (16.6) | <0.001 |
| Dabigatran | 24 (5.9) |  | 24 (13.7) | <0.001 |
| Treatment combinations, n(%) | | | | |
| DAPT combinations | | | | |
| Aspirin + clopidogrel |  | 204 (89.4) |  | |
| Aspirin + prasugrel |  | 17 (7.5) |  |  |
| Aspirin + ticagrelor |  | 7 (3.1) |  |  |
| Patients in anticoagulants + SAPT | | | | |
| Acenocoumarol + aspirin |  | | 38 (9.4) |  |
| Acenocoumarol + clopidogrel |  |  | 8 (2) |  |
| Acenocoumarol + ticagrelor |  |  | 0 (0) |  |
| Acenocoumarol + prasugrel |  |  | 0 (0) |  |
| DOAC + aspirin |  |  | 14 (3.5) |  |
| DOAC + clopidogrel |  |  | 27 (6.7) |  |
| DOAC + ticagrelor |  |  | 0 (0) |  |
| DOAC + prasugrel |  |  | 0 (0) |  |
| Patients in triple treatment | | | | |
| Any triple treatment (OAC + DAPT) |  | | 81 (46.3) |  |
| VKA + DAPT |  |  | 49 (28) |  |
| DOAC + DAPT |  |  | 32 (18.3) |  |

# Supplementary table 3. Outcomes

|  | **DAPT**  **(n=228)** | **Anticoagulants ± antiplatelets**  **(n=175)** | **p-value** | **HR (95%CI)** | **p-value^2^** |
| --- | --- | --- | --- | --- | --- |
| **Efficacy, composite** n (%) | | | | | |
| Composite of all-cause mortality, reinfarction, stroke | 33 (14.5) | 23 (13.1) | 0.809 | 0.847 (0.49 - 1.44) | 0.543 |
| **Efficacy, components of the composite outcome**, n (%) | | | | | |
| All-cause mortality | 10 (4.4) | 5 (2.9) | 0.422 | 0.63 (0.22 - 1.86) | 0.407 |
| Reinfarction | 26 (11.4) | 20 (11.6) | 0.961 | 0.93 (0.53 - 1.67) | 0.817 |
| Stroke | 2 (0.9) | 3 (1.7) | 0.458 | 1.82 (0.3 -10.93) | 0.610 |
| **Bleeding, composite outcome** | | | | | |
| Bleeding | 25 (11) | 25 (14.3) | 0.316 | 1.25 (0.72 - 2.19) | 0.422 |
| **Bleeding, components of the composite outcome**, n (%) | | | | | |
| GUSTO mild | 9 (4) | 29 (11.4) | 0.004 | 2.77 (1.27 - 6.1) | 0.011 |
| GUSTO moderate | 15 (6.58) | 2 (1.2.29) | 0.044 | 0.33 (0.11 - 1.01) | 0.053 |
| GUSTO severe or life-threatening | 1 (0.44) | 0 (0) | 0.380 | - | - |

#

# Kaplan Meier Survival Analyses


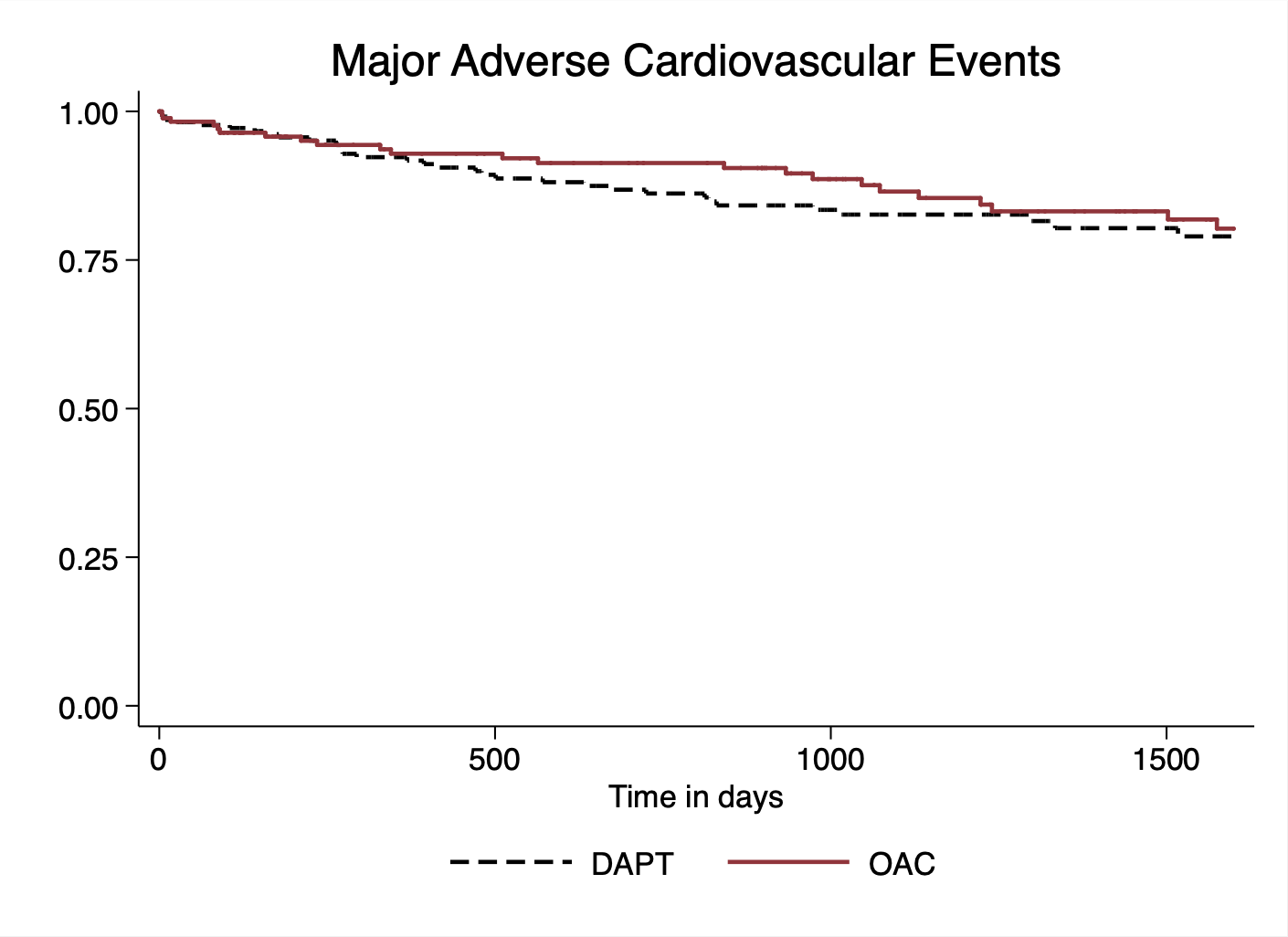


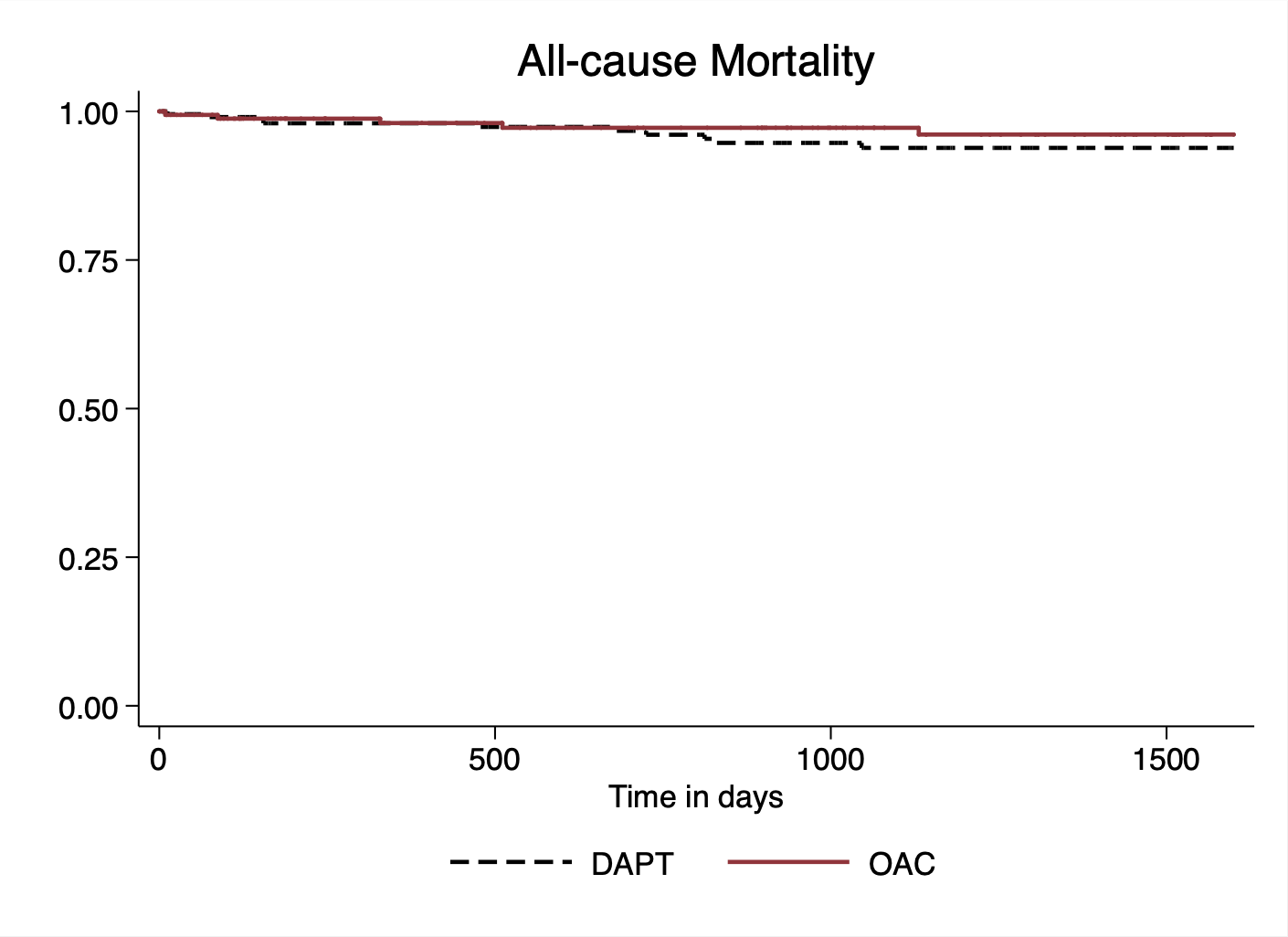


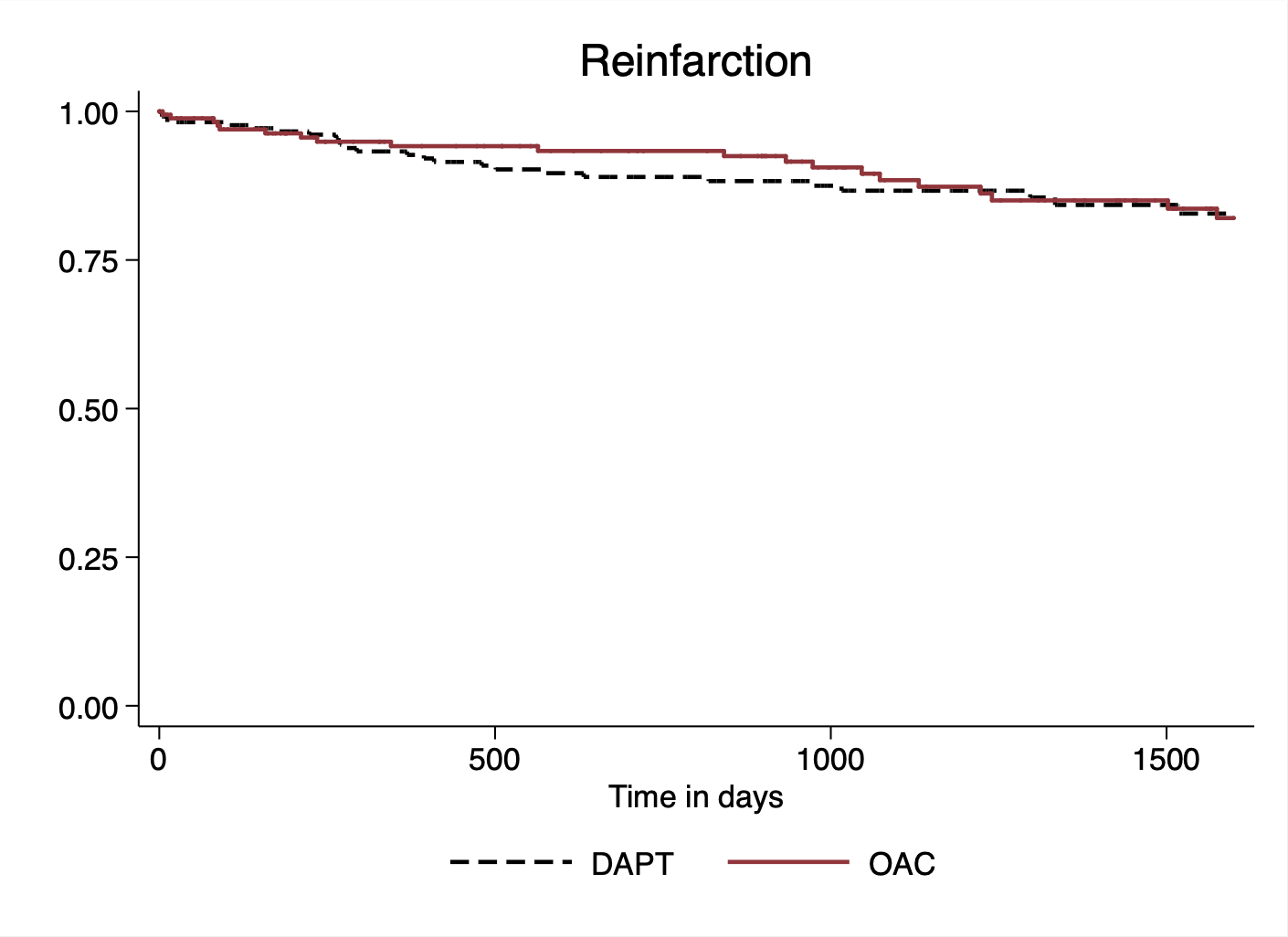


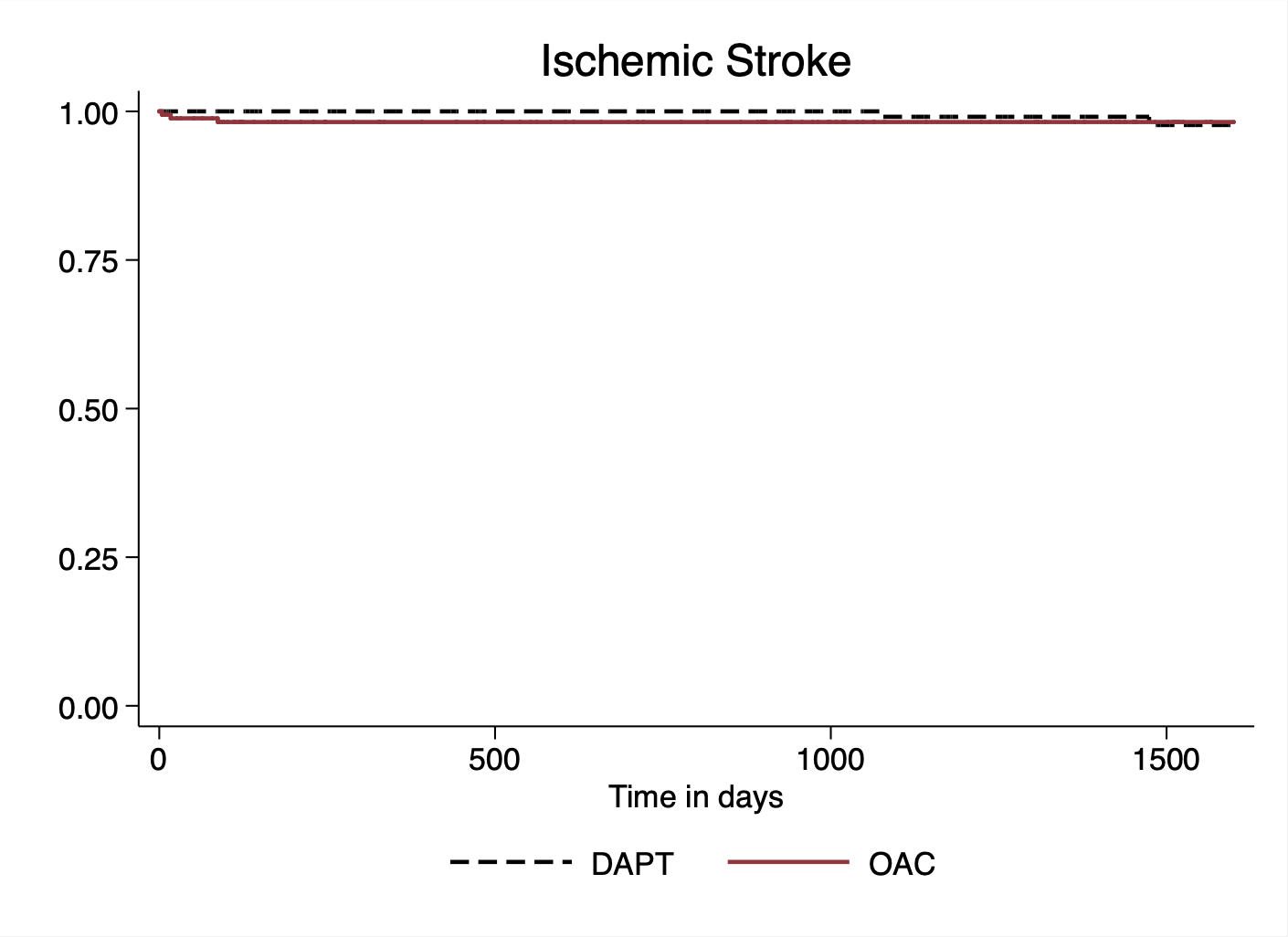


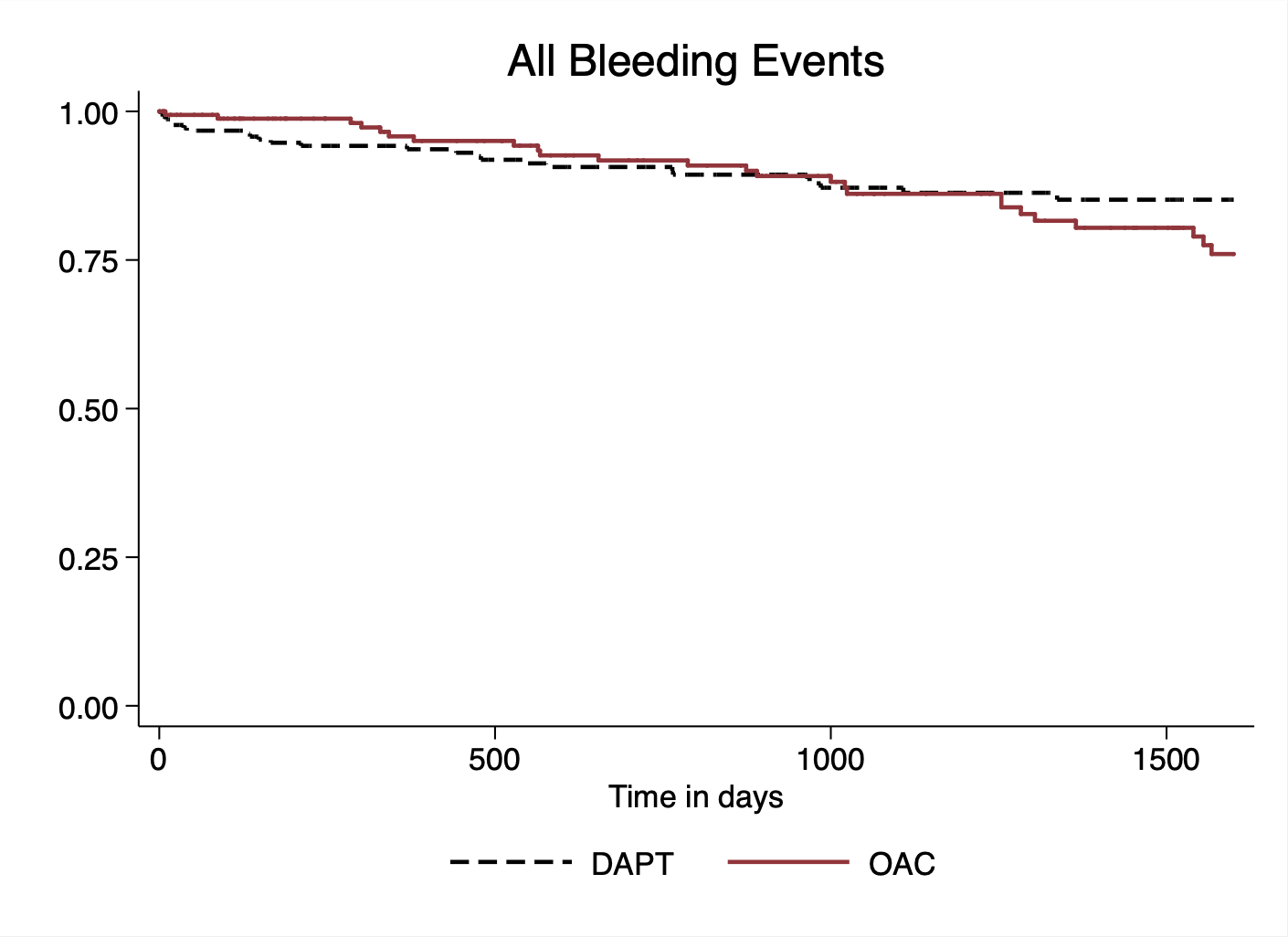


# 
