# Supplementary material for: Anticoagulant versus Antiplatelet Therapy After Acute Coronary Syndromes in Patients with Coronary Artery Ectasia: A Retrospective Cohort Study
Source: Cardiovasc Drugs Ther. 2025 Sep 24;40(3):1021–33. doi: 10.1007/s10557-025-07784-0 (PMC13171775; doi:10.1007/s10557-025-07784-0)

**Propensity Score Matching Analysis**

**Table 1.** Logistic regression for treatment used.

|  | **Univariate analysis** | | **Multivariate adjusted analysis** | |
| --- | --- | --- | --- | --- |
|  | **OR (95% CI)** | **p-value** | **OR (95% CI)** | **p-value** |
| *Demographic characteristics* | | | | |
| Male sex | 0.96 (0.49 - 1.88) | 0.901 |  |  |
| Age ≥ median (58) | 0.90 (0.58 - 1.39) | 0.624 |  |  |
| Hypertension | 1.01 (0.65 - 1.58) | 0.948 |  |  |
| Type 2 diabetes | **0.44 (0.24 - 0.80)** | **0.008** |  |  |
| Tobacco Use | 1.09 (0.70 - 1.71) | 0.701 |  |  |
| BMI ≥ 30 | **1.61 (1.02 - 2.54)** | **0.040** |  |  |
| Markis ≤2 (High grade) | **4.2 (2.44 - 7.25)** | **<0.001** | **3.38 (1.88 - 6.06)** | **<0.001** |
| *Ectasia in culprit artery location:* | | | | |
| Left main coronary | **1.94 (1.17 - 3.23)** | **0.010** |  |  |
| Left descending   - **Diffuse LAD** | **2.76 (1.65 - 4.62)**   - **2.49 (1.47 - 4.22)** | **<0.001**   - **0.001** |  |  |
| Circumflex   - Diffuse LCX | **3.09 (1.92 - 4.97)**   - **2.86 (1.77 - 4.63)** | **<0.001**   - **<0.001** |  |  |
| Right coronary   - Diffuse RCA | **2.02 (1.07 - 3.85 )**   - 1.32 (0.85 - 2.06) | **0.031**   - 0.214 |  |  |
| *Clinical presentation and treatment* | | | | |
| STEMI | **1.97 (1.22 - 3.19)** | **0.006** | **2.31 (1.36 - 3.95)** | **0.002** |
| Stenting | **0.22 (0.14 - 0.36)** | **<0.001** | **0.27 (0.16 - 0.46)** | **<0.001** |
| TIMI after stenting ≥2 | **0.43 (0.24 - 0.75)** | **0.003** |  |  |
| TMP ≥2 | **0.41 (0.22 - 0.76)** | **0.005** |  |  |
| LVEF at discharge below 50% | 1.16 (0.74 - 1.81) | 0.514 |  |  |

**Table 2.** Baseline characteristics of the matched population.

|  | **Total Population**  **(n=244)** | **DAPT**  **(n=122)** | **Anticoagulants ± antiplatelets**  **(n=122)** | **p-value** |
| --- | --- | --- | --- | --- |
| **Demographics** | | | | |
| Male sex, *n ( %)* | 210 (86.1) | 103 (84.4) | 107 (87.7) | 0.460 |
| Age, *mean (SD)* | 57.7 (±10.7) | 57.7 (±11.2) | 57.7 (±10.2) | 0.958 |
| BMI, *median (IQR)* | 28.1 (25.5 - 31.6) | 27.4 (24.5 - 30.8) | 28.7 (27.3 - 32.4) | <0.001 |
| **Comorbidities, *n (%)*** | | | | |
| Hypertension | 141 (57.8) | 71 (58.2) | 70 (57.4) | 0.897 |
| Type 2 diabetes | 49 (20.2) | 33 (27.3) | 16 (13.1) | 0.006 |
| Tobacco use | 141 (57.8) | 68 (56.2) | 73 (59.8) | 0.566 |
| **CAE characteristics** | | | | |
| Markis Classification, *n (%)* | | | | |
| Markis 1 and 2 (High grade) | 197 (80.7) | 95 (77.9) | 102 (83.6) | 0.256 |
| Markis 3 and 4 (Low grade) | 47 (19.3) | 27 (22.1) | 20 (16.4) |  |
| Location, *n (%)* | | | | |
| Left main coronary | 67 (27.5) | 29 (23.8) | 38 (31.2) | 0.197 |
| Left descending artery   - Diffuse LAD | 183 (75.6)   - 64 (26.2) | 86 (71.7)   - 26 (21.3) | 97 (79.5)   - 38 (31.2) | 0.155   - 0.081 |
| Left circumflex artery   - Diffuse LCX | 158 (65.3)   - 87 (35.7) | 70 (58.3)   - 35 (28.7) | 88 (72.1)   - 52 (42.6) | 0.024   - 0.023 |
| Right coronary artery   - Diffuse RCA | 212 (87.6)   - 125 (51.2) | 104 (86.7)   - 63 (51.6) | 108 (88.5)   - 62 (50.8) | 0.661   - 0.898 |
| **Presentation, *n (%)*** | | | | |
| STEMI | 178 (73) | 88 (72.1) | 90 (73.8) | 0.773 |
| NSTEMI | 53 (21.7) | 27 (22.1) | 26 (21.3) | 0.877 |
| Unstable angina | 13 (5.3) | 7 (5.7) | 6 (4.9) | 0.776 |
| **Clinical Scores** | | | | |
| GRACE, *median (IQR)* | 110 (87 - 134) | 107 (87 - 130) | 112 (91 - 139) | 0.214 |
| Killip-Kimball≥II, *n (%)* | 56 (23) | 29 (23.8) | 27 (22.1) | 0.761 |
| TIMI, *n (%)*   - 0-2 - 3-7 - ≥8 | - 98 (40.2) - 141 (57.8) - 5 (2.1) | - 46 (37.7) - 72 (59) - 4 (3.3) | - 52 (42.6) - 69 (56.6) - 1 (0.8) | - 0.513 - 0.795 - 0.175 |
| CRUSADE, *median (IQR)* | 22 (16 - 32) | 24 (16 - 34) | 21 (17 - 29) | 0.497 |
| CHA_2_DS_2_-VASc≥2*, n (%)* | 141 (57.8) | 74 (60.7) | 67 (54.9) | 0.436 |
| HAS-BLED≥2*, n (%)* | 114 (46.7) | 63 (51.6) | 51 (41.8) | 0.158 |
| **Treatment, *n (%)*** | | | | |
| Thromboaspiration | 32 (13.2) | 13 (10.7) | 19 (15.6) | 0.266 |
| GpIIa/IIIb inhibitor | 90 (36.9) | 41 (33.9) | 49 (40.2) | 0.377 |
| Stenting | | | | |
| Stent in any artery | 81 (33.2) | 48 (39.3) | 33 (27.1) | 0.041 |
| Stent in the culprit artery | 73 (29.9) | 45 (37.2) | 28 (23) | 0.010 |
| Stent in other arteries | 18 (7.4) | 10 (8.2) | 8 (6.6) | 0.611 |
| TIMI after stenting ≥2*, n (%)* | 194 (79.5) | 103 (84.4) | 91 (74.6) | 0.057 |
| TMP ≥2*, n (%)* | 201 (82.4) | 105 (86.1) | 96 (78.7) | 0.130 |
| **Follow up** | | | | |
| Follow-up in days**,** *median (IQR)* | 1046.5 (303 - 1759.5) | 1004 (264 - 1684) | 1111 (335 - 1799) | 0.506 |

CAE: coronary artery ectasia, CKD: chronic kidney disease, DAPT: double antiplatelet therapy, GFR: glomerular filtration rate, IQR: interquartile range, LCX: left circumflex artery, LAD: left descending artery, LVEF: left ventricular ejection fraction, NSTEMI: non-ST elevation myocardial infarction, RCA: right coronary artery, SD: standard deviation, STEMI: ST-elevation myocardial infarction.

**Table 3.** Outcome analysis with matched population.

|  | **DAPT**  **(n=122)** | **Anticoagulants ± antiplatelets**  **(n=122)** | **p-value** | **HR (95%CI)** | **p value^2^** |
| --- | --- | --- | --- | --- | --- |
| **Efficacy, composite** n (%) | | | | | |
| Composite of all-cause mortality, reinfarction, stroke | 21 (17.2%) | 15 (12.3%) | 0.279 | 0.65 (0.34 - 1.26) | 0.204 |
| **Efficacy, components of the composite outcome**, n (%) | | | | | |
| All-cause mortality | 7 (5.7%) | 1 (0.8%) | 0.031 | 0.14 (0.02 - 1.12) | 0.064 |
| Reinfarction | 16 (13.1%) | 13 (10.7%) | 0.569 | 0.74 (0.35 - 1.53) | 0.413 |
| Stroke | 0 (0%) | 2 (1.6%) | 0.159 | ---- | --- |
| **Bleeding, composite outcome** | | | | | |
| Bleeding | 17 (13.9%) | 18 (14.8%) | 0.855 | 1.08 (0.59 - 1.96) | 0.806 |

# *CI: confidence interval, DAPT: double antiplatelet therapy, HR: hazard ratio.*

**Figure 1.** Bias of relevant variables after propensity score matching.


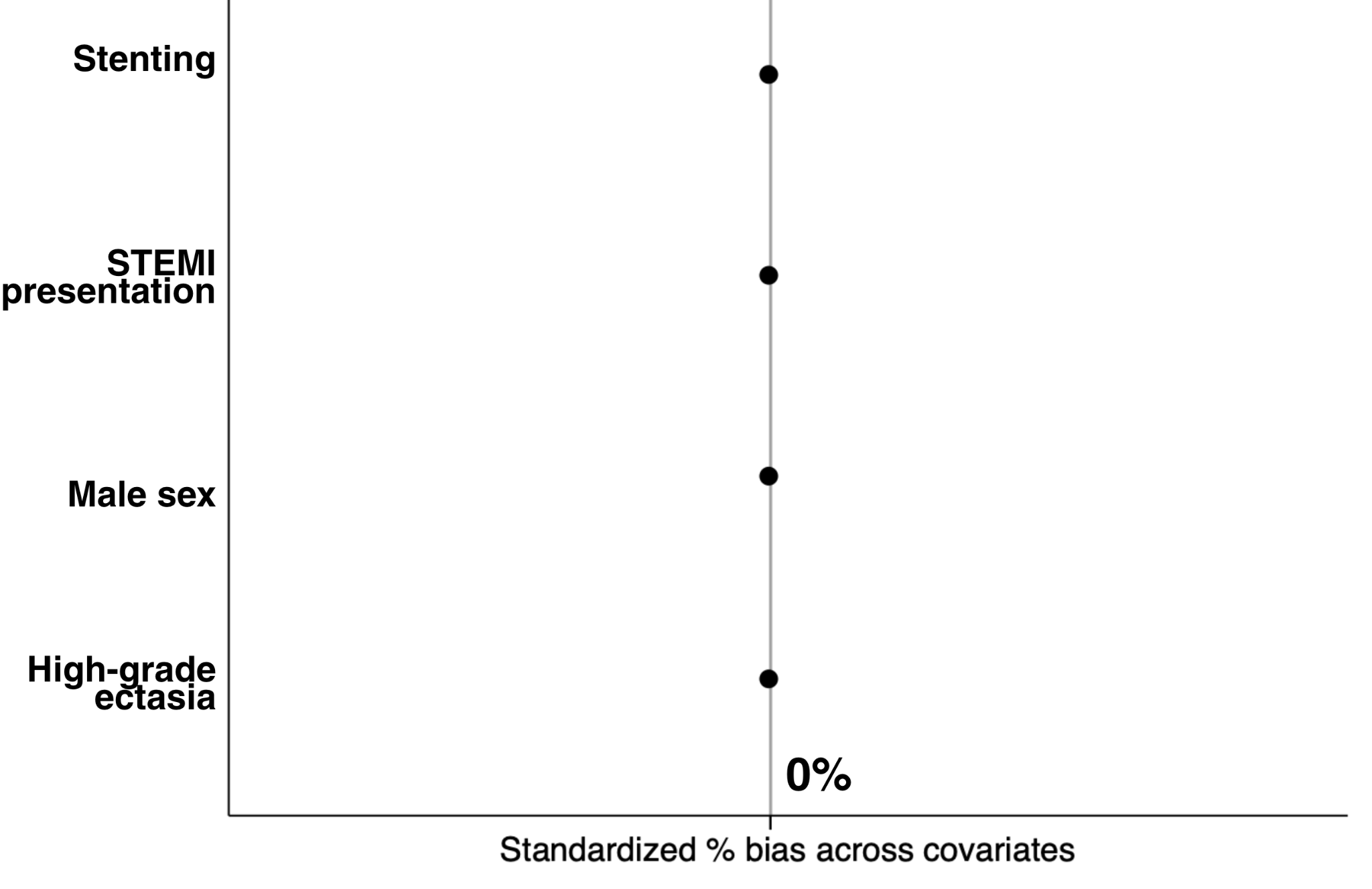


**Figure 2.** Kaplan Meier Curve of the primary component in matched population.


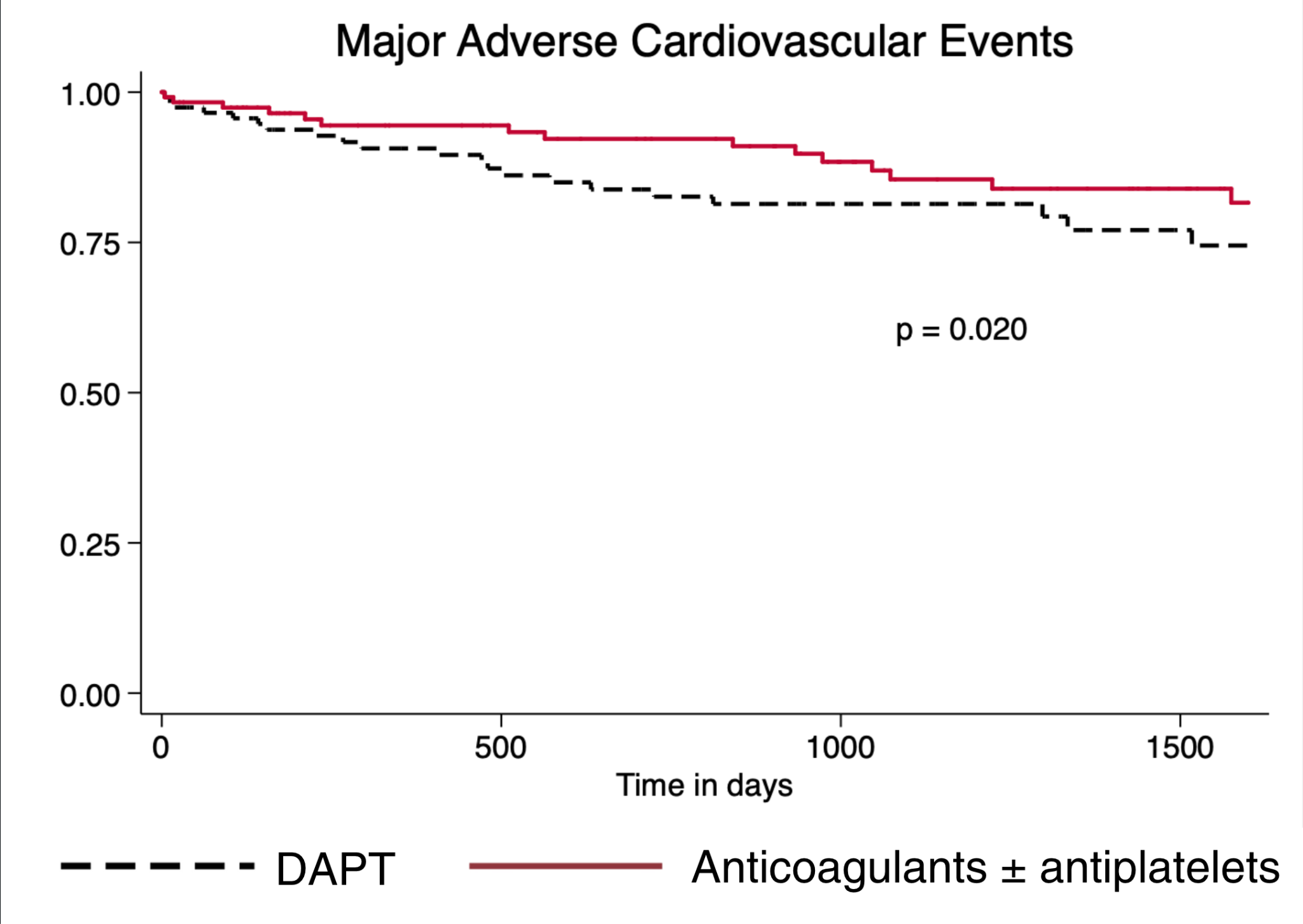

Supplement: Supplementary file 3 — Supplementary file3 (DOCX 308 KB) [file 10557_2025_7784_MOESM3_ESM.docx]
